# NRF2 Plays a Critical Role in Both Self and EGCG Protection against Diabetic Testicular Damage

**DOI:** 10.1155/2017/3172692

**Published:** 2017-06-18

**Authors:** Chenyu Pan, Shengzhu Zhou, Junduo Wu, Lingyun Liu, Yanyan Song, Tie Li, Lijuan Ha, Xiaona Liu, Fuchun Wang, Jingyan Tian, Hao Wu

**Affiliations:** ^1^Department of Anesthesiology, The Second Hospital of Jilin University, 218 Ziqiang St., Changchun, Jilin 130041, China; ^2^Department of Cardiology, The Second Hospital of Jilin University, 218 Ziqiang St., Changchun, Jilin 130041, China; ^3^Department of Andrology, The First Hospital of Jilin University, 71 Xinmin St., Changchun, Jilin 130021, China; ^4^Department of Nephrology, The Second Hospital of Jilin University, 218 Ziqiang St., Changchun, Jilin 130041, China; ^5^Department of Acupuncture and Tuina, Changchun University of Chinese Medicine, 1035 Boshuo Rd., Changchun, Jilin 130117, China; ^6^The '973' National Basic Research Program of China, Changchun University of Chinese Medicine, 1035 Boshuo Rd., Changchun, Jilin 130117, China; ^7^Department of Urology, Second Division of The First Hospital of Jilin University, 3302 Jilin Rd., Changchun, Jilin 130031, China

## Abstract

Activation of nuclear factor erythroid 2-related factor 2 (NRF2) has been found to ameliorate diabetic testicular damage (DTD) in rodents. However, it was unclear whether NRF2 is required for these approaches in DTD. Epigallocatechin gallate (EGCG) is a potent activator of NRF2 and has shown beneficial effects on multiple diabetic complications. However, the effect of EGCG has not been studied in DTD. The present study aims to explore the role of NRF2 in both self and EGCG protection against DTD. Therefore, streptozotocin-induced diabetic C57BL/6 wild type (WT) and *Nrf2* knockout (KO) mice were treated in the presence or absence of EGCG, for 24 weeks. The *Nrf2* KO mice exhibited more significant diabetes-induced loss in testicular weight and spermatozoa count, and increase in testicular apoptotic cell death, as compared with the WT mice. EGCG activated NRF2 expression and function, preserved testicular weight and spermatozoa count, and attenuated testicular apoptotic cell death, endoplasmic reticulum stress, inflammation, and oxidative damage in the WT diabetic mice, but not the *Nrf2* KO diabetic mice. The present study demonstrated for the first time that NRF2 plays a critical role in both self and EGCG protection against DTD.

## 1. Introduction

Diabetes causes damage to multiple organs, including testis [1]. Decreased sperm cell count and velocity were found in patients with diabetes [2]. Moreover, diabetics had increased sperm nuclear and mitochondrial DNA damage [3], along with increased level of advanced glycation end products (AGEs) in the testis, epididymis, and sperm [4]. Diabetes-induced excessive AGEs can cause oxidative stress, leading to activation of mitochondria or endoplasmic reticulum (ER) stress-related cell death pathways, the effect of which may result in sperm loss [5–8]. Therefore, targeting diabetes-induced oxidative stress is a promising strategy to prevent male infertility in diabetic patients.

Nuclear factor erythroid 2-related factor 2 (NRF2) is a master factor in the cellular antioxidant system [9, 10]. NRF2 activates the transcription of a cohort of antioxidant genes, such as heme oxygenase-1 (*Ho1*) and NAD(P)H dehydrogenase quinone 1 (*Nqo1*) [11], the proteins of which work as scavengers of diabetes-induced free radicals. Notably, the *Nrf2* gene knockout (KO) male mice developed infertility in an age-dependent manner [12]. NRF2 and its downstream antioxidants were found to be expressed in the male reproductive tract and played a critical role in defence against oxidative stress [12, 13]. Furthermore, low *Nrf2* mRNA was found to be associated with impaired sperm function parameters in human individuals, including concentration, motility, vitality, and morphology [14]. All these findings suggested that NRF2 plays a pivotal protective role in male infertility. Approaches in activating NRF2, such as administration of the NRF2 activator sulforaphane (SFN) [5, 6], supplementation of zinc [15], and exposure to low-dose X-irradiation [8], effectively ameliorated diabetes-induced testicular inflammation, ER stress, and apoptotic cell death, in rodent models of both type 1 and type 2 diabetes. However, it was unclear whether NRF2 was required for the protective effect of these approaches.

Epigallocatechin gallate (EGCG) is the most abundant and effective green tea catechin and is known to be a potent NRF2 activator [16–20]. Despite showing beneficial effects in a variety of diseases [21–24], EGCG has not been studied for its effect in diabetic testicular damage (DTD). It was also not previously known whether NRF2 is required for this possible action of EGCG. Therefore, the present study aims to answer the following questions: (1) whether or not EGCG has a protective role in ameliorating DTD and (2) whether or not NRF2 is required for self and EGCG protection against DTD. To these ends, diabetes was induced in 8-week-old male C57BL/6 wild-type (WT) and *Nrf2* KO mice by streptozotocin (STZ). Both the diabetic and nondiabetic mice were treated in the presence or absence of EGCG, for a total period of 24 weeks.

## 2. Methods

### 2.1. Animal Treatment

C57BL/6 WT (*Nrf2*+/+) and *Nrf2* KO (*Nrf2*−/−) mice were obtained through breeding of heterozygotes (*Nrf2*+/−) [10, 25]. All mice were housed in the Animal Center of Changchun University of Chinese Medicine at 22°C, on a 12 : 12-hour light-dark cycle with free access to rodent feed and tap water. The Institutional Animal Care and Use Committee at Changchun University of Chinese Medicine approved all the experimental procedures, which were consequently in accordance with the International Guiding Principles for Biomedical Research Involving Animals, as issued by the Council for the International Organizations of Medical Sciences. Eight-week-old male mice received either sodium citrate or STZ (Sigma-Aldrich, St. Louis, MO; 50 mg/kg daily, dissolved in 0.1 M sodium citrate, pH 4.5) through intraperitoneal injection for 5 consecutive days. Fasting glucose levels (4-hour fast) were measured a week after the last injection of STZ. Mice with a fasting glucose level higher than 250 mg/dl were considered diabetic. In order to observe the chronic effect of diabetes, the mice were kept for 24 weeks post diabetes onset. Thus, the diabetic mice and age-matched controls were treated daily by subcutaneously injected EGCG (100 mg/kg [26], ≥98%, dissolved in normal saline, PureOne Biotechnology, Shanghai, PRC) or normal saline daily, for a total period of 24 weeks. Blood glucose was recorded on days 0, 28, 56, 84, 112, 140, and 168, after diabetes onset. The mice were then euthanized under anaesthesia by intraperitoneal injection of chloral hydrate at 0.3 mg/kg [27]. The testes and cauda epididymides were harvested for analysis. Six mice in each group were studied.

### 2.2. Sperm Density Assessment

The cauda epididymis from each mouse was placed in 2 ml Earle's balanced salt solution (Sigma-Aldrich) supplemented with 0.1% bovine serum albumin (Sigma-Aldrich). The epididymis was gently teased with a bent needle to release spermatozoa under observation through a stereomicroscope (Olympus). Sperm density was assessed with a haemocytometer and was presented by spermatozoa count per epididymis [28–30].

### 2.3. Hematoxylin and Eosin (H&E) Staining, Terminal Deoxynucleotidyl Transferase-Mediated dUTP Nick End Labeling (TUNEL) Assay, and Immunohistochemical (IHC) Staining

Testes were fixed immediately in 10% buffered formalin solution after harvesting and were embedded in paraffin and sectioned into 5 *μ*m thick sections onto glass slides. The sections were processed for H&E staining and TUNEL staining, as previously described [6]. For TUNEL staining, apoptotic cells exhibited a brown nuclear stain as TUNEL positive cells and were counted manually under a microscope. Four sections from each testis were counted. On each section, 30 seminiferous tubule's cross sections from each testis were selected in the same pattern, moving each slide without repetitive counting in a blinded fashion [31]. Results were presented as TUNEL positive cells per 10^3^ cells. For IHC staining, the sections were incubated with anti-3-nitrotyrosine (3-NT, Millipore, Temecula, CA, USA, 1 : 100) overnight at 4°C. After washing with PBS, the sections were incubated with horseradish peroxidase-conjugated secondary antibody (Santa Cruz Biotechnology, Dallas, TX, USA, 1 : 300 dilutions in PBS) for 2 hours at room temperature. The sections were then treated with peroxidase substrate 3,3′-diaminobenzidine in the developing system provided by Vector Laboratories (Burlingame, CA, USA) and counterstained with hematoxylin.

### 2.4. Reactive Oxygen Species (ROS) Assay

Testicular ROS generation was measured using a ROS assay kit provided by Nanjing Jiancheng Bioengineering Institute (Nanjing, Jiangsu, PRC), following the manufacturer's instructions.

### 2.5. Isolation of Nuclei

Nuclei were isolated from testicular tissue of each mouse using a nuclei isolation kit provided by Sigma-Aldrich, following the manufacturer's instructions, as previously described [25, 30, 32].

### 2.6. Western Blot Analysis

Western blot was performed using testicular tissue as described in our previous study [33]. The primary antibodies included anti-activating transcription factor 4 (ATF4, Cell Signaling Technology, Danvers, MA, USA, 1 : 1000), anti-Bcl-2-associated X protein (Bax, Cell Signaling Technology, 1 : 1000), anti-B-cell lymphoma 2 (Bcl-2, Santa Cruz Biotechnology, 1 : 2000), anti-binding immunoglobulin protein (BIP, Cell Signaling Technology, 1 : 1000), anti-caspase12 (Cell Signaling Technology, 1 : 1000), anti-cleaved caspase3 (c-caspase3, Cell Signaling Technology, 1 : 1000), anti-C/EBP homologous protein (CHOP, Cell Signaling Technology, 1 : 1000), anti-GAPDH (Santa Cruz Biotechnology, 1 : 2000), anti-histone H3 (Santa Cruz Biotechnology; 1 : 1000), anti-intercellular adhesion molecule 1 (ICAM-1, Santa Cruz Biotechnology, 1 : 500), anti-inducible nitric oxide synthase (iNOS, Cell signaling Technology, 1 : 1000), anti-NRF2 (Santa Cruz Biotechnology, 1 : 1000), anti-pro-caspase3 (Cell Signaling Technology, 1 : 1000), and anti-vascular cell adhesion molecule 1 (VCAM-1, Santa Cruz Biotechnology, 1 : 500).

### 2.7. Quantitative Reverse Transcription PCR (qPCR)

qPCR was performed as previously described [34, 35]. Primers for *Ho1* (product number: Mm00516005_m1) and *Nqo1* (product number: Mm01253561_m1) were purchased from Life Technologies (Grand Island, NY, USA). Thermal cycling was carried out as the following: 95°C for 3 minutes (m) as initial denaturing, 45 cycles at 94°C for 30 seconds (s), 60°C for 30 s, and 72°C for 60 s, followed by a final extension at 72°C for 2 m.

### 2.8. Quantitative Analysis of Lipid Peroxides

Testicular malondialdehyde (MDA) concentration was measured using a lipid peroxidation assay kit (Sigma-Aldrich), following the manufacturer's instructions, as previously described [6].

### 2.9. Statistical Analysis

Six mice in each group were researched. Indices in each group were measured and summarized as means ± SD. Image Quant 5.2 (GE Healthcare Bio-Sciences, Pittsburgh, PA, USA) was used to analyse the density of Western blot images. IHC positive area was quantified by the Image-Pro Plus Version 6.0 software (Media Cybernetics, Rockville, MD, USA). One-way ANOVA was performed for comparisons among different groups, followed by post hoc pairwise comparisons using Tukey's test with Origin 8.6 data analysis and graphing software Lab (OriginLab, Northampton, MA, USA). A test was significant if *p* < 0.05.

## 3. Results

### 3.1. Deletion of the *Nrf2* Gene Completely Abrogated Both Self and EGCG Protection against Diabetes-Induced Testicular Weight Loss and Reduction in Spermatozoa Count

**Figure 1:**
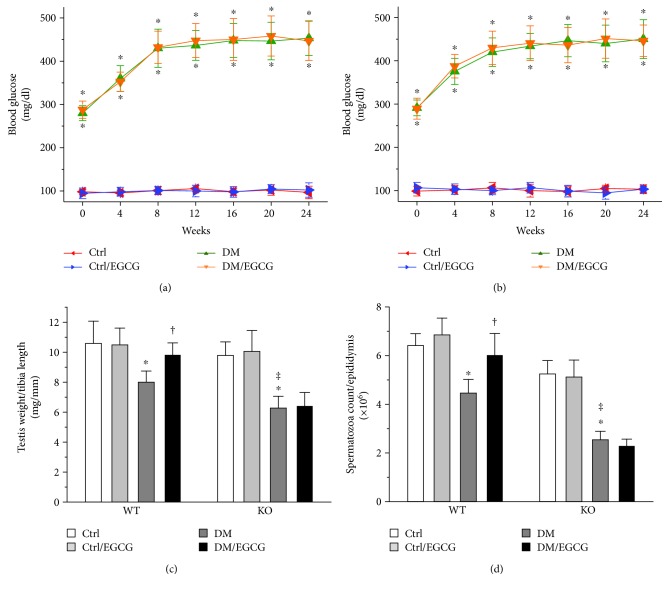
Deletion of the *Nrf2* gene completely abrogated both self and EGCG protection against diabetes-induced testicular weight loss and reduction in spermatozoa count. Diabetes was induced in 8-week-old male C57BL/6 WT and *Nrf2* KO mice by streptozotocin. Blood glucose was monitored in both the (a) WT and (b) *Nrf2* KO mice at the multiple time points 0, 4, 8, 12, 16, 20, and 24 weeks post diabetes onset. (c) Testis weight to tibia length ratio and (d) spermatozoa count were calculated at the time, 24 weeks post diabetes onset, at which the mice were killed. Data were presented as means ± SD (*n* = 6). ^∗^*p* < 0.05 versus Ctrl; ^†^*p* < 0.05 versus DM; ^‡^*p* < 0.05 versus WT DM. WT: wild type; KO: knockout; Ctrl: control; DM: diabetes mellitus.

Both the WT and the *Nrf2* KO diabetic mice developed significantly higher blood glucose levels at 0, 4, 8, 12, 16, 20, and 24 weeks post diabetes onset, as compared with respective controls (Figures 1(a) and 1(b)). EGCG had no impact on blood glucose levels in either type of the mice, under either diabetic or nondiabetic conditions (Figures 1(a) and 1(b)). Diabetes caused a significant decrease in the ratio of testis weight to tibia length and spermatozoa count in either type of the mice (Figures 1(c) and 1(d)). Notably, the *Nrf2* KO diabetic mice suffered from more marked decrease in the two indices, as compared with the WT diabetic mice (Figures 1(c) and 1(d)). The WT diabetic mice, but not the *Nrf2* KO diabetic mice, were protected by EGCG from diabetes-induced reduction in testicular weight and spermatozoa count (Figures 1(c) and 1(d)). These findings suggested that NRF2 plays a critical role in both self-protection and EGCG protection against diabetes-induced loss in testis weight and spermatozoa count.

### 3.2. NRF2 Was Required for Both Self and EGCG Protection against Diabetes-Induced Testicular Apoptotic Cell Death

**Figure 2:**
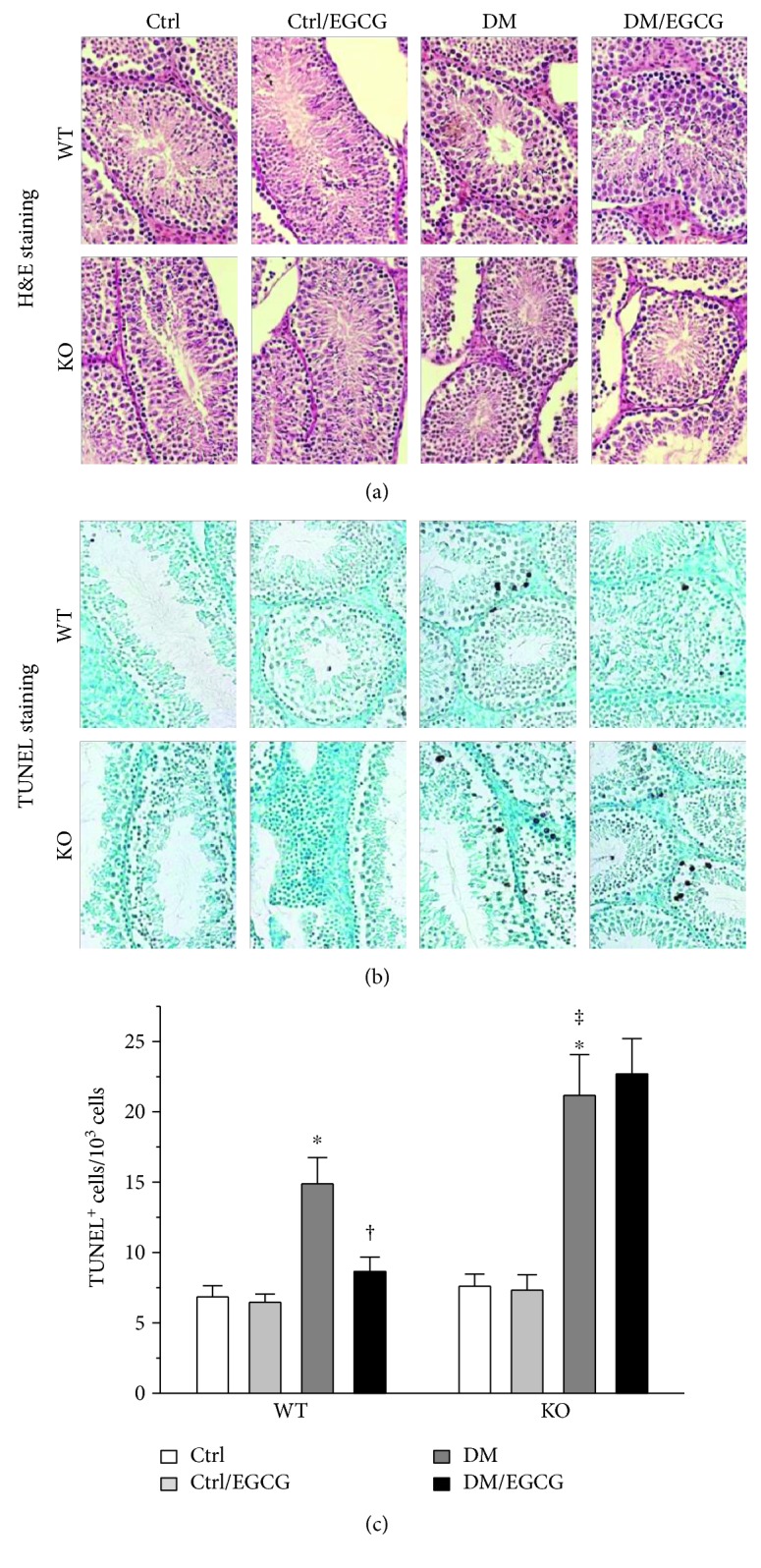
NRF2 was required for both self and EGCG protection against diabetes-induced testicular apoptotic cell death. (a) H&E staining was conducted for observation of morphological change. (b) Testicular apoptotic cell death was detected by TUNEL assay, from which (c) TUNEL^+^ cells were calculated. Data were presented as means ± SD (*n* = 6). ^∗^*p* < 0.05 versus Ctrl; ^†^*p* < 0.05 versus DM; ^‡^*p* < 0.05 versus WT DM. H&E: hematoxylin and eosin; TUNEL: terminal deoxynucleotidyl transferase-mediated dUTP nick end labelling; ^+^: positive; other abbreviations are the same as those in Figure 1.

Diabetes did not lead to obvious testicular pathological changes, as presented by H&E staining (Figure 2(a)). However, apoptotic cell death was prominent in the diabetic testes of either type of the mice, as shown by TUNEL staining (Figure 2(b)). Notably, diabetes-induced testicular apoptotic cell death was more significant in the *Nrf2* KO mice, as compared to the WT mice (Figure 2(c)). EGCG significantly decreased the number of testicular TUNEL positive cells in the WT diabetic mice, but not the *Nrf2* KO diabetic mice (Figure 2(c)).

### 3.3. EGCG Prevented Diabetes-Induced Activation of Testicular Apoptotic Cell Death Signaling via NRF2

**Figure 3:**
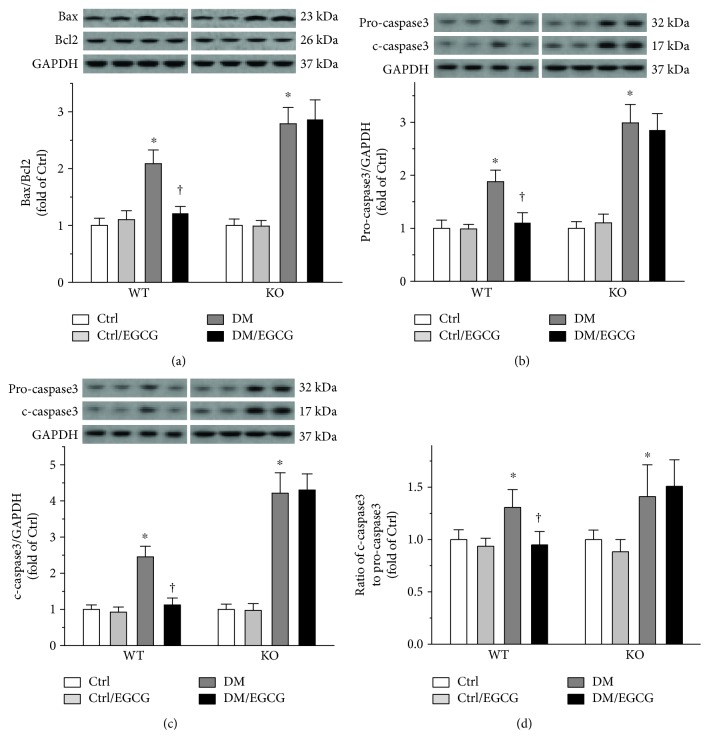
EGCG prevented diabetes-induced activation of testicular apoptotic cell death signaling via NRF2. Testicular apoptotic signaling was further evaluated by determining (a) the ratio of Bax protein level to Bcl2 protein level, along with the protein levels of (b) pro-caspase3 and (c) c-caspase3. To further assess the activity of caspase3, (d) the ratio of c-caspase3 to pro-caspase3 was calculated. For (b) and (c), the protein levels were normalized with GAPDH. Data were normalized as fold variation to Ctrl and were presented as means ± SD (*n* = 6). ^∗^*p* < 0.05 versus Ctrl; ^†^*p* < 0.05 versus DM. Bax: Bcl-2-associated X protein; Bcl-2: B-cell lymphoma 2; c-caspase3: cleaved caspase3; other abbreviations are the same as those in Figure 1.

The status of testicular apoptotic cell death was further confirmed by determining the ratio of Bax to Bcl2 (Bax/Bcl2, Figure 3(a)) and the protein levels of pro-caspase3 and c-caspase3 (Figures 3(b) and 3(c)). Bax/Bcl2 and the protein levels of pro-caspase3 and c-caspase3 were all significantly elevated in the testes of the diabetic mice (Figures 3(a), 3(b), and 3(c)), the effects of which were almost completely prevented by EGCG in the WT mice (Figures 3(a), 3(b), and 3(c), left panels). However, deletion of the *Nrf2* gene completely abrogated these efficacies of EGCG (Figures 3(a), 3(b), and 3(c), right panels). To further evaluate caspase3 activity, the ratio of c-caspase3 to pro-caspase3 was calculated in all groups and comparisons were constructed between the groups (Figure 3(d)). As shown in Figure 3(d), EGCG prevented the diabetes-elevated ratio of c-caspase3 to pro-caspase3 (Figure 3(d), left panel) in the WT mice, but not in the *Nrf2* KO mice (Figure 3(d), right panel).

### 3.4. NRF2 Mediated EGCG Prevention of Diabetes-Induced Testicular ER Stress

**Figure 4:**
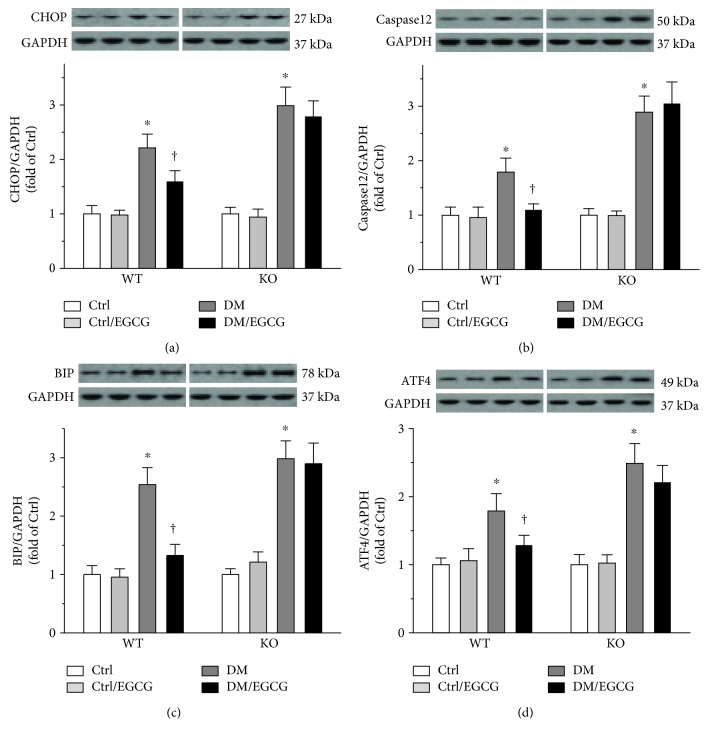
NRF2 mediated EGCG prevention of diabetes-induced testicular ER stress. The status of ER stress was determined by measuring protein levels of (a) CHOP, (b) caspase12, (c) BIP, and (d) ATF4, using Western blot. The protein levels were normalized with GAPDH. Data were normalized as fold variation to Ctrl and were presented as means ± SD (*n* = 6). ^∗^*p* < 0.05 versus Ctrl; ^†^*p* < 0.05 versus DM. ER: endoplasmic reticulum; CHOP: C/EBP homologous protein; BIP: binding immunoglobulin protein; ATF4: activating transcription factor 4; other abbreviations are the same as those in Figure 1.

ER stress was evaluated by determining the protein levels of CHOP (Figure 4(a)), caspase12 (Figure 4(b)), BIP (Figure 4(c)), and ATF4 (Figure 4(d)), all of which were elevated in the diabetic testes. EGCG decreased these indices in the WT, but not the *Nrf2* KO, diabetic mice (Figures 4(a), 4(b), 4(c), and 4(d)). The results indicated NRF2 to be the key factor through which EGCG prevented diabetes-induced testicular ER stress.

### 3.5. EGCG Completely Lost the Efficacy in Ameliorating Diabetes-Induced Testicular Inflammation and Oxidative Damage in the Absence of NRF2

**Figure 5:**
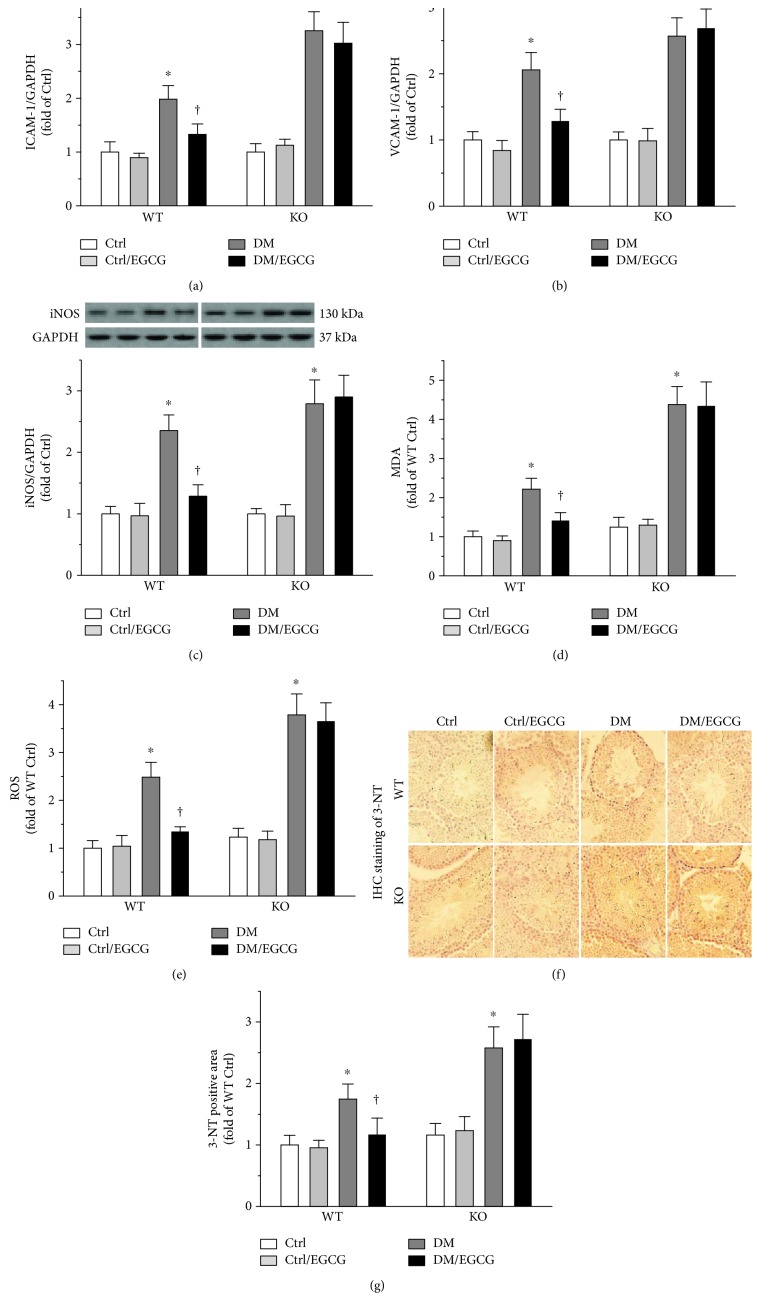
EGCG completely lost the efficacy in ameliorating diabetes-induced testicular inflammation and oxidative damage in the absence of NRF2. Testicular inflammation was assessed by determining protein levels of (a) ICAM-1 and (b) VCAM-1, using Western blot. To determine testicular oxidative stress, (c) iNOS protein was determined by Western blot. The protein levels were normalized with GAPDH. Data were normalized as fold variation to Ctrl and were presented as means ± SD (*n* = 6). To further evaluate testicular oxidative stress, ELISAs were performed to detect (d) MDA and (e) ROS levels, and (f) IHC staining for 3-NT was also performed. Data were normalized as fold variation to WT Ctrl and were presented as means ± SD (*n* = 6). ^∗^*p* < 0.05 versus Ctrl; ^†^*p* < 0.05 versus DM. ICAM-1: intercellular adhesion molecule 1; VCAM-1: vascular cell adhesion molecule 1; iNOS: inducible nitric oxide synthase; MDA: malondialdehyde; ROS: reactive oxygen species; IHC: immunohistochemical; 3-NT: 3-nitrotyrosine; other abbreviations are the same as those in Figure 1.

Testicular inflammation was evaluated by determining protein levels of ICAM-1 (Figure 5(a)) and VCAM-1 (Figure 5(b)). Testicular oxidative damage was determined by measuring iNOS protein level (Figure 5(c)), MDA level (Figure 5(d)), and ROS generation (Figure 5(e)). In the WT mice, EGCG markedly decreased these diabetes-elevated indices (Figures 5(a), 5(b), 5(c), 5(d), and 5(e), left panels). These effects of EGCG were completely lost in the absence of NRF2 (Figures 5(a), 5(b), 5(c), 5(d), and 5(e), right panels). The status of testicular oxidative stress was further evaluated by immunohistochemical staining of 3-NT (Figure 5(f)), an indicator of oxidative/nitrosative damage. As shown in Figure 5(g), EGCG completely lost the protective role in attenuating the diabetes induction of 3-NT in the absence of NRF2.

### 3.6. EGCG Enhanced Testicular NRF2 Expression and Function

**Figure 6:**
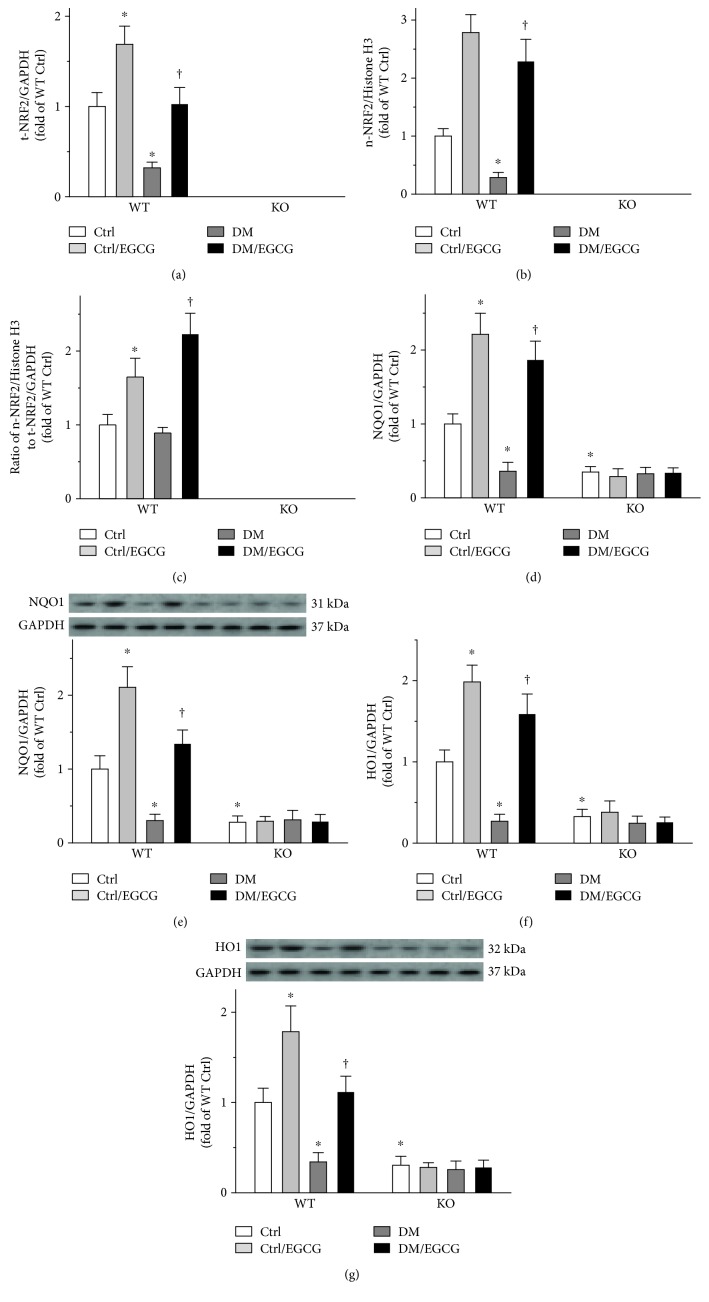
EGCG enhanced testicular NRF2 expression and function. Testicular (a) t-NRF2 and (b) n-NRF2 protein were determined by Western blot in all the mice. (c) The ratio of n-NRF2 to t-NRF2 was calculated to indicate NRF2 nuclear translocation. To evaluate NRF2 function, the expression of *Nqo1* and *Ho1* were further determined, by measuring *Nqo1* (d) mRNA and (e) protein levels, as well as *Ho1* (f) mRNA and (g) protein levels. t-NRF2 protein and the expression of *Nqo1* and *Ho1* were normalized to GAPDH. n-NRF2 was normalized to Histone H3. Data were normalized as fold variation to WT Ctrl and were presented as means ± SD (*n* = 6). ^∗^*p* < 0.05 versus WT Ctrl; ^†^*p* < 0.05 versus WT DM. t-NRF2: total NRF2; n-NRF2: nuclear NRF2; *Nqo1*: NAD(P)H dehydrogenase quinone 1; *Ho1*: heme oxygenase-1; other abbreviations are the same as those in Figure 1.

Testicular whole cell NRF2 (total NRF2, t-NRF2) and nuclear NRF2 (n-NRF2) were both increased by EGCG in the WT mice, under either diabetic or nondiabetic conditions (Figures 6(a) and 6(b), left panels). NRF2 protein was not detectable in the testes of the *Nrf2* KO mice (Figures 6(a) and 6(b), right panels), the result of which confirmed the deletion of the *Nrf2* gene. The ratio of n-NRF2/Histone H3 to t-NRF2/GAPDH (Figure 6(c)) was calculated to reflect the proportion of NRF2 that translocated to the nucleus. This ratio was found to be increased by EGCG in either diabetic or nondiabetic WT mice (Figure 6(c), left panel). In order to evaluate NRF2 function, the expression of *Nqo1* (Figures 6(d) and 6(e)) and *Ho1* (Figures 6(f) and 6(g)) was determined. As shown in Figures 6(d), 6(e), 6(f), and 6(g), the mRNA and protein levels of *Nqo1* and *Ho1* were all elevated by EGCG in the WT mice (Figures 6(d), 6(e), 6(f), and 6(g), left panels), but not in the *Nrf2* KO mice (Figures 6(d), 6(e), 6(f), and 6(g), right panels). Moreover, the *Nrf2* KO mice had lower basal expression of *Nqo1* and *Ho1*, as compared with the WT mice (Figures 6(d), 6(e), 6(f), and 6(g)).

## 4. Discussion

The present study determined the effect of EGCG on the prevention of DTD. The results showed that diabetes caused significant testicular weight loss, decreased spermatozoa count, and increased testicular apoptotic cell death, ER stress, and oxidative damage, as compared with control. Notably, these detrimental outcomes were more prominent in the *Nrf2* KO mice, as compared with the WT mice. EGCG activated NRF2 signaling and produced a significant attenuation of the testicular damage caused by diabetes in the WT mice. However, deletion of the *Nrf2* gene completely abolished the protective effect of EGCG.

Oxidative stress is considered to be one of the main mechanisms through which diabetes causes long-term complications [36–38]. Significant oxidative damage was observed in the testes of diabetic mice [5–8, 15]. Given that NRF2 plays a critical role in cellular defence against diabetes-induced oxidative stress, approaches to activate testicular NRF2 have been tested in diabetic mice, including administration of the NRF2 activator SFN [5, 6], supplementation of zinc [15], and exposure to low-dose X-irradiation [8]. Although the effects of the approaches were promising, it was still unclear whether NRF2 activation is required for the protective effect of the approaches. In the present study, by using *Nrf2* KO mice, NRF2 was found to be the key factor through which EGCG ameliorated DTD. In addition, enhanced oxidative stress status was observed in a rat model of prediabetes [39, 40], and white tea consumption restored sperm quality in the prediabetic rats by ameliorating testicular oxidative damage [40]. The present study supports the previous report by Oliveira et al. [40], with an emphasis on the long-term DTD.

One novel finding of the present study was the protective role of NRF2 in self-prevention of the pathogenesis of DTD (Figures 1(c), 1(d), 2(b), and 2(c)), in addition to the finding that NRF2 was required for the protective effect of EGCG on DTD. The self-protective role of NRF2 observed in the present study is in accordance with the previous findings which showed that NRF2 played a key preventive role in diabetic cardiomyopathy [41] and nephropathy [32, 42, 43]. The beneficial role of NRF2 in multiple organs or systems under diabetic condition [38] may support the use of NRF2 activators, even though the activators may not be specific to an organ, tissue, or cell type.

NRF2 activators have been developed based on different regulatory mechanisms. Zinc was reported to upregulate NRF2 protein in the testes of diabetic rats [15], although the mechanism by which zinc increased NRF2 was not investigated. The finding that zinc enhanced NRF2 expression and function via activating protein kinase B- (PKB- or AKT-) mediated inhibition of Fyn function [44] might provide a clue for the possible mechanism by which zinc activated NRF2 in the testes of diabetic rats. Low-dose radiation was also recently reported to attenuate testicular apoptosis in diabetic rats [8]. The study indicated that low-dose radiation inhibited protein tyrosine phosphatase-1B and tribbles homologue 3, the effect of which resulted in AKT-mediated activation of testicular NRF2 signaling [8]. Therefore, zinc and low-dose radiation shared the same AKT signaling pathway to activate testicular NRF2. SFN is a potent NRF2 activator. Kelch-like ECH-associated protein 1 (KEAP1) is the key negative cytoplasmic regulator of NRF2 [11, 45]. SFN activates NRF2 signaling by modulating the structure of KEAP1 protein, resulting in the release of NRF2 from the KEAP1-NRF2 complex [11, 45]. Although previous studies showed that SFN activated NRF2 and ameliorated diabetes-induced testicular apoptotic cell death without knowing the expression of *Keap1* [5, 6], we speculate that inhibition of KEAP1 function by SFN could be the mechanism through which SFN activated NRF2 in these studies. Similar to SFN, EGCG is also known to activate NRF2 by inactivating KEAP1 [46, 47]. EGCG is speculated to directly interact with the cysteine residues present in KEAP1, thereby stimulating NRF2 dissociation from KEAP1 [48]. However, another study indicated that EGCG might induce NRF2 via activation of AKT and ERK in human mammary epithelial cells [18]. Future studies are needed to elucidate the exact mechanisms of EGCG and other NRF2-activating approaches in the regulation of NRF2 in DTD.

The NRF2 activator SFN has already been tested in several clinical trials [49]. Furthermore, the approval of dimethyl fumarate (BG-12), another NRF2 activator, for use in the treatment of multiple sclerosis [50] is the confirmation of NRF2 being a viable drug target in disease. However, to date, no NRF2 activator has been applied in clinical trials for DTD or diabetes-induced male infertility. Hence, attention should be paid to the critical role of NRF2 in this diabetic complication.

Taken together, the present study demonstrates, for the first time, that NRF2 plays a key role in self and EGCG protection against diabetic testicular damage. Therefore, this study may provide a basis for potential application of EGCG or other NRF2 activators in future clinical trials.
